# T Lymphocyte Integrated Endoplasmic Reticulum Ca^2+^ Store Signaling Functions Are Linked to Sarco/Endoplasmic Reticulum Ca^2+^-ATPase Isoform-Specific Levels of Regulation

**DOI:** 10.3390/ijms26094147

**Published:** 2025-04-27

**Authors:** Md Nasim Uddin, David W. Thomas

**Affiliations:** Department of Pharmaceutical Sciences, Thomas J. Long School of Pharmacy, University of the Pacific, Stockton, CA 95211, USA; m_uddin1@u.pacific.edu

**Keywords:** calcium homeostasis, T cell signaling, SERCA, calcium pumps, ER calcium stores, calcium signaling

## Abstract

We explored the effects of altering expression levels of the sarco/endoplasmic reticulum Ca^2+^-ATPase (SERCA) ion-transporting enzymes on key T lymphocyte signaling functions. In these studies, we have taken advantage of the Jurkat T cell line which provides a T lymphocyte model cell phenotype with a well-characterized T cell receptor (TCR)-activated signaling pathway, as well as offering a cellular system with a good understanding of the SERCA expression profile. These studies have been prompted by a strong imperative to gain a better understanding of the complex roles SERCA Ca^2+^ pumps play in the integrated TCR-activated signaling network, particularly given the central role of SERCA functions in regulating essential endoplasmic reticulum (ER) integrity. We find in this study that altering SERCA expression can significantly reconfigure ER Ca^2+^ stores, increasing or decreasing Ca^2+^ storage capacity depending on upregulation or downregulation of SERCA expression, and these effects are also associated with substantial changes in agonist-induced Ca^2+^ release and influx patterns. Not surprisingly, these fundamental changes in TCR-regulated Ca^2+^ signaling properties are associated with major alterations in T lymphocyte functions including regulation of growth patterns, cytokine secretion and energy utilization. Our study also describes additional evidence revealing intriguing functional distinctions between the major SERCA isoform-regulated Ca^2+^ stores in T lymphocytes. Our work thus serves to reinforce increasing efforts to target the SERCA pumps as a potential profitable strategy to produce novel engineered T lymphocytes in the rapidly growing field of T-cell immunotherapy

## 1. Introduction

T lymphocytes are the central coordinators of the adaptive immune system, amplifying specific antigen recognition into a multi-cellular deployment involving recruitment of antibody-secreting and pathogen-eliminating lymphocyte populations [1]. Given their preeminent role in managing immune responses, there is a compelling need to understand the details underlying the signaling pathways activated by antigen recognition that precede and are essential to mobilizing effective immune responses. The T cell receptor (TCR) complex is the molecular structure that transduces specific antigen recognition into multiple complex intracellular signaling networks resulting in coordinated gene expression, cell activation and proliferation, culminating in the production of a panoply of high-efficacy differentiated effector phenotypes [2,3,4,5].

A prominent central nodal point of the TCR-activated signaling pathway is the Ca^2+^ signal, which is, in turn, tightly regulated at multiple sites by the high affinity sarco-endoplasmic reticulum (ER) Ca^2+^-ATPase (SERCA) enzymes. The SERCA Ca^2+^ transporters or pumps sit at a pivotal site presiding over the integrity of the multiplex ER functions, including integrated regulation of TCR-activated Ca^2+^ signals, as well as, importantly, ER-derived signals that influence T lymphocyte fate via production of either cell survival or apoptotic factors [4,6,7,8].

Thus, the elucidation of complex SERCA pump functions within these dynamic signaling environments is essential and may enable modulatory alterations of SERCA function via genetic or pharmacologic means to stabilize and sustain critical T cell functions in either immunodeficient or hyper-immunologic states. We were thus motivated to investigate effects of altered SERCA pump expression, using the often-used T cell line model Jurkat T lymphocyte, to gain further insight into SERCA regulation of key T lymphocyte signaling functions [9,10,11,12].

We report findings in this study that underscore the expanding and integrated roles SERCA Ca^2+^ pumps play in key T lymphocyte functions; thus, we observed substantial changes in Ca^2+^ storage, release and influx patterns with altered SERCA expression levels that also translated to significant regulatory effects on cytokine synthesis and glucose metabolism, factors likely embedded in the larger aggregate effects of SERCA function on T lymphocyte growth control. These findings have important implications for the increasing efforts to engineer T lymphocytes with favorable phenotypic features that confer greater functional resilience in disease immunopathologic states such as cancer and autoimmunity [13,14,15].

## 2. Results

### 2.1. Overexpression of the SERCA 2b and SERCA 3 Ca^2+^ ATPase Pumps Alters T Cell Ca^2+^ Signaling Pathways, Remodeling Both Ca^2+^ Release and Influx Responses

We sought to examine the hypothesis that altering specific SERCA pump isoform functions, employing genetic methods to both overexpress and reduce protein expression, could rapidly and significantly reconfigure Ca^2+^ signaling patterning in T cells.

**Figure 1:**
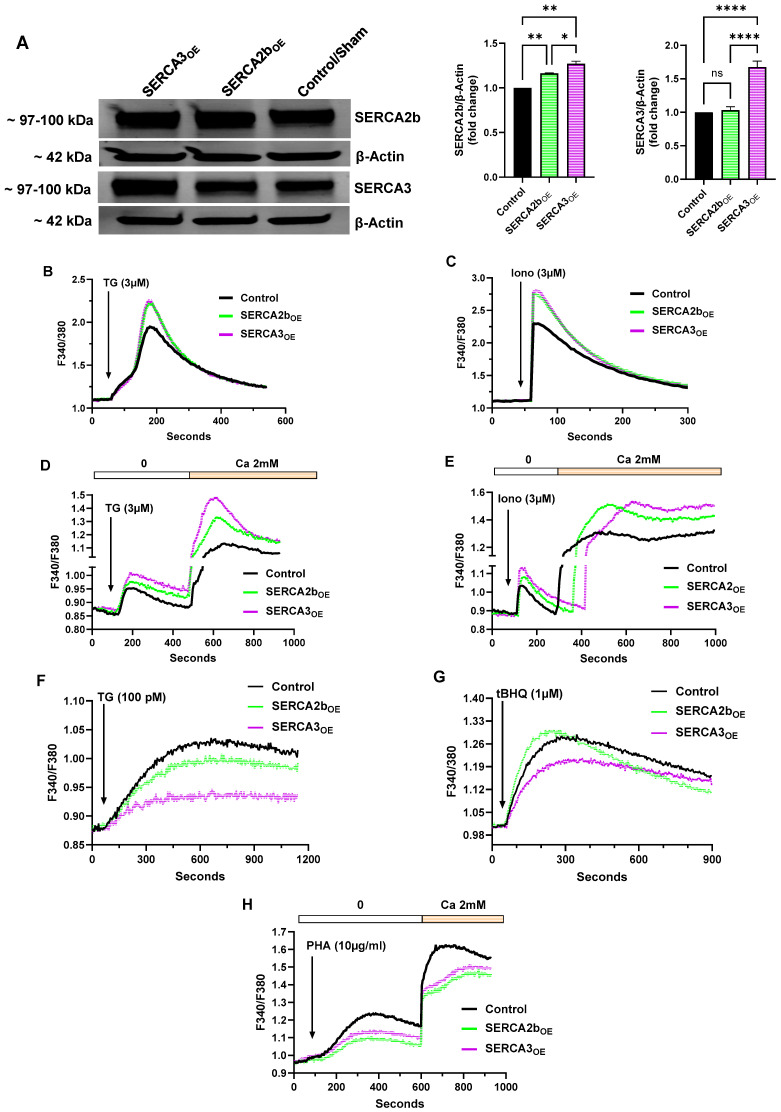
Overexpression of SERCA 2b and SERCA 3 Ca^2+^-ATPases Alters Ca^2+^ Signaling Properties in Jurkat T Lymphocytes. (**A**) Representative Western blot image of overexpression of SERCA 2b and SERCA 3 Ca^2+^-ATPase isoforms in Jurkat lymphocytes (see Section 4). The figure shows the approximate molecular weight of the SERCA proteins and β-actin protein bands as control. Additionally shown are the densitometry bar plots derived from band density quantification for SERCA 2b (green bars) and SERCA 3 (purple bars) compared to control sham transfected cells (black bars). (**B**,**C**) Jurkat T lymphocytes were loaded with Fura-2 and suspended in Ca^2+^-free media (balanced salt solution plus 2 mM EGTA) in cuvettes for use in the large cell-number configuration (1 × 10^6^ cells/mL). (**B**) Jurkat cell Ca^2+^ release responses in SERCA 2b_OE_, SERCA 3_OE_ and control cells induced by the addition of TG (3 μM, arrow) as determined by the ratio of fluorescence changes at 340 and 380 nm (F340/380). (**C**) the same experiment as in *B* but with the application of ionomycin (3 μM, arrow). (**D**,**E**) Ca^2+^ measurements were acquired from Jurkat T lymphocytes in the small cell-number configuration (30–60 cells per field) using microscope photometry (Section 4). (**D**) Jurkat cell Ca^2+^ release and influx responses in SERCA 2b_OE_, SERCA 3_OE_ and control cells induced by the addition of TG (3 μM, arrow); Ca^2+^ influx responses were determined by the addition of Ca^2+^ (2 mM) to the coverslips as indicated. (**E**) the same experiment as in *D* but with the application of ionomycin (3 μM, arrow). (**F**,**G**) Jurkat T lymphocytes were loaded with Fura-2 and suspended in Ca^2+^-free media (balanced salt solution plus 2 mM EGTA) in cuvettes for use in the large-cell number configuration (1 × 10^6^ cells/mL) and challenged with the low-dose SERCA blocker regimen. (**F**) Jurkat cell Ca^2+^ release responses in SERCA 2b_OE_, SERCA 3_OE_ and control cells induced by the addition of low-dose TG (100 pM, arrow) as determined by the ratio of fluorescence changes at 340 and 380 nm (F340/380). (**G**) the same experiment as in *F* but with the application of low-dose tBHQ (1 μM, arrow). (**H**), Jurkat cell Ca^2+^ release and influx responses in SERCA 2b_OE_, SERCA 3_OE_ and control cells induced by the addition of PHA (10 μg/mL, arrow); Ca^2+^ influx responses were determined by the addition of Ca^2+^ (2 mM) to the cuvettes as indicated. Fluorescence traces of Ca^2+^ experiments shown are representative of three to six separate experiments with significant differences assessed via use of student t test. One-way ANOVA followed by Tukey’s multiple comparison tests were used to analyze Western blot data for SERCA expression levels with n = 3 (Section 4). Asterisks denote statistical significance with * *p* < 0.05, ** *p* < 0.005, **** *p* < 0.0001 and *ns* is not significant *p* > 0.05.

Figure 1A shows protein overexpression of the SERCA 2b and SERCA 3 Ca^2+^-ATPase pumps, the two dominant Ca^2+^ pump isoforms expressed in T cells as well as the Jurkat lymphocyte [16,17], displaying both the Western blot image and bar graphs of band intensities. Jurkat lymphocytes were transfected via electroporation (Neon NxT electroporation system) with plasmid constructs containing sequences encoding ATP2A2 (SERCA 2b) and ATP2A3 (SERCA 3), and transfections consistently yielded between 85–95% efficiencies, as assessed by co-transfection of a GFP-encoding plasmid, (SERCA 2b expression levels were 115–120% of control, and similarly, SERCA 3 expression levels were 165–170% of control sham transfected Jurkat lymphocytes). Interestingly, we observed an apparent linkage in these experiments between increased SERCA 3 expression and a concomitant increase in SERCA 2b expression (Figure 1A), whereas the opposite effect was not observed, given that increased SERCA 2b expression did not appear to enhance SERCA 3 expression. These results suggest that SERCA expression levels may not be independent events in cellular Ca^2+^ homeostasis, with SERCA 2b expression levels exhibiting biased sensitivity to changes in SERCA 3 pump expression.

Jurkat cells overexpressing either SERCA 2b (SERCA2b_OE_) or SERCA 3 (SERCA3_OE_) were then tested for responses to an array of Ca^2+^ mobilizing agonists in both large and small-cell population studies. In most experiments, Ca^2+^ release responses were measured while cells were suspended in nominally Ca^2+^-free media; in some experiments, however, Ca^2+^ influx was determined by adding Ca^2+^ back to cells in suspension. Moreover, most experiments were conducted using large cell-number populations (approximately 1 × 10^6^ cells/mL) in cuvette-based spectrofluorimetry; where indicated, we also conducted Ca^2+^ measurements using microscope fluorimetry, sampling responses from small cell-number populations (approximately 30–60 cells).

Figure 1B shows a clear increase in Ca^2+^ store levels, as determined by peak height fluorescent changes as well as Ca^2+^ release rates, in cells exposed to thapsigargin (TG, 3 μM) in both SERCA 2b_OE_ (peak height ΔF 1.14 ± 0.03 fluorescence ratio units and Ca^2+^ release rate ΔF/sec 9.5 × 10^−3^, n = 5, green trace) and SERCA 3_OE_ (ΔF 1.16 ± 0.05 fluorescence ratio units and Ca^2+^ release rate ΔF/sec 9.5 × 10^−3^, n = 5, purple trace) cells vs. control cells (ΔF 0.85 ± 0.06 fluorescence ratio units and Ca^2+^ release rate ΔF/sec 7.1 × 10^−3^, n = 5, black trace). The high dose of TG used in these experiments likely reflect aggregate Ca^2+^ levels stored in all SERCA-regulated Ca^2+^ pools; given at this concentration of TG, all SERCA pumps will be inhibited. Similarly, Figure 1C shows Ca^2+^ release responses elicited by high levels of the Ca^2+^ ionophore ionomycin (Iono, 3 μM), with a similar effect in exhibiting a significantly greater increase in peak height amplitudes in both SERCA 2b_OE_ (ΔF 1.64 ± 0.05 fluorescence ratio units and Ca^2+^ release rate ΔF/sec 7.5 × 10^−2^, n = 5, green trace) and SERCA 3_OE_ (ΔF 1.69 ± 0.04 fluorescence ratio units and Ca^2+^ release rate ΔF/sec 7.7 × 10^−2^, n = 5, purple trace) Jurkat lymphocytes compared to control (ΔF 1.19 ± 0.07 fluorescence ratio units and Ca^2+^ release rate ΔF/sec 5.4 × 10^−2^, n = 5, black trace). The high concentration of ionomycin used in this experiment serves to capture the global status of Ca^2+^ stores given Ca^2+^ release will occur in both TG-sensitive as well as TG-insensitive Ca^2+^ pools. These experiments suggest that overexpression of the SERCA Ca^2+^ pumps in Jurkat T cells results in greater Ca^2+^ storage capacity, presumably due to increased Ca^2+^ pump activity.

Figure 1D,E show experimental results that are a repeat of the experiments shown in Figure 1B,C, with the exception that these experiments were performed using our small cell-number Ca^2+^ measurements, with an additional assessment of Ca^2+^ influx responses induced by stimulus application in Ca^2+^ add-back experiments. We were prompted to verify that our responses shown in Figure 1B,C in Jurkat large cell-number population measurements exposed to high concentrations of Ca^2+^-mobilizing agonists were representative and could be captured using higher resolution measurements employing microscope photometry methods collecting fluorescence emission from small cell-number samples (30–60 cells). Figure 1D,E show that high dose TG (3 μM) and ionomycin (3 μM) induce the same general response in small cell-number populations as was observed in large cell-number population experiments (Figure 1B,C, 2 × 10^6^ cells), revealing again that SERCA 2b_OE_ and SERCA 3_OE_ result in apparently greater Ca^2+^ storage capacity in T cells. These experiments using small cell-number populations, however, did reveal slightly greater sample resolution, allowing a bit finer distinction in the Ca^2+^ release responses in SERCA 2b_OE_ and SERCA 3_OE_ cells, respectively. We observed, for example, increased Ca^2+^ release to high dose TG and ionomycin in SERCA 3_OE_ cells compared to SERCA 2b_OE_ cells (purple trace vs. green trace). We noted above that overexpression of the SERCA 3 Ca^2+^ pump was also associated with increased SERCA 2b expression (Figure 1A); thus, the observed enhanced Ca^2+^ release in SERCA 3_OE_ Jurkat lymphocytes may be attributable to greater overall SERCA pump expression and activity, resulting in increased Ca^2+^ storage compared to SERCA 2b_OE_ cells (Figure 1D,E).

Given the well-known tight coupling between T cell Ca^2+^ stores and Ca^2+^ influx pathways [2,4,18], we examined the effects of SERCA pump overexpression on TG- and ionomycin-induced Ca^2+^ influx responses. As shown in Figure 1D,E, we observed significantly increased Ca^2+^ influx responses when Ca^2+^ was added back following TG- and ionomycin-induced store depletion, respectively (Figure 1D, TG response: ΔF 0.41 ± 0.03 SERCA2b_OE_ and ΔF 0.53 ± 0.05 SERCA3_OE_ vs. ΔF 0.25 ± 0.03 control cells, n = 3, *p* < 0.05; Figure 1E, ionomycin response: ΔF 0.62 ± 0.02 SERCA2b_OE_ and ΔF 0.61 ± 0.04 SERCA3_OE_ vs. ΔF 0.44 ± 0.05 control cells, n = 3, *p* < 0.05). These experiments utilize aggressive Ca^2+^ store depletion methods with high dose TG and ionomycin, both suggesting that increasing SERCA2b or SERCA 3 pump expression in T lymphocytes significantly augments the capacity of cells to activate and sustain store-depletion-coupled Ca^2+^ entry pathways. Thus, these experiments suggest that T lymphocytes may be able to modulate SERCA pump expression levels, enabling recruitment of tailored Ca^2+^ influx responses appropriately scaled to antigen-activated pathways of variable strength.

The experiments shown in Figure 1B–E suggest that SERCA overexpression can alter the status of T lymphocyte Ca^2+^ stores and, via increased pump activity, expand Ca^2+^ storage capacity. We next sought to investigate whether SERCA overexpression could influence Ca^2+^ signals elicited at a finer level of stimulus strength more in line with a physiological agonist, using cuvette spectrofluorimetry with large cell-number populations to more accurately measure the smaller amplitude signals induced by these agents. We chose to use the low-dose SERCA blocker strategy that our previous work has shown enables assessment of Ca^2+^ release from specific SERCA 2b and SERCA 3 Ca^2+^ stores in T lymphocytes [19]. Figure 1F,G show experiments challenging Jurkat lymphocytes with very low TG concentrations (100 pM), which allows specific disruption of SERCA 2b stores ([19], Figure 1F) and also low concentrations of 2,5-di-(tert butyl)-1,4-benzohydroquinone (tBHQ), which correspondingly exerts preferential disruption of SERCA 3 Ca^2+^ stores (Figure 1G). Figure 1F shows that overexpression of the SERCA Ca^2+^-ATPases yielded intriguing pump isoform-specific differences when the Jurkat lymphocytes were exposed to the low TG concentrations. In contrast to the increased Ca^2+^release responses shown in Figure 1B,D to high TG levels (3 μM), we observed that SERCA 2b_OE_ cells displayed reduced Ca^2+^ release activity to treatment with much lower TG levels (100 pM, ΔF 0.13 ± 0.003 and Ca^2+^ release rate ΔF/sec 2.7 × 10^−4^ for SERCA2b_OE_ vs. ΔF 0.16 ± 0.004 and Ca^2+^ release rate ΔF/sec 3.35 × 10^−4^ for control cells n = 3, *p* < 0.05). Surprisingly, this effect was even greater in the SERCA 3_OE_ Jurkat cells, revealing a greater curtailment of Ca^2+^ release than was observed in SERCA 2b_OE_ and control cells (Figure 1F, ΔF 0.07 ± 0.002 and Ca^2+^ release rate ΔF/sec 1.46 × 10^−4^ for SERCA3_OE_ vs. ΔF 0.16 ± 0.004 and Ca^2+^ release rate ΔF/sec 3.35 × 10^−4^ for control cells n = 3, *p* < 0.05).

These results suggest that when T lymphocytes are exposed to a minimal or manageable SERCA pump stress challenge, increasing expression of either SERCA 2b or SERCA 3 confers sufficient pump function to offset and contain Ca^2+^ release induced by nearly subthreshold TG stimulation. Indeed, overexpression of the SERCA 3 isoform appears to produce a T lymphocyte phenotype with the greatest capacity to access increased pump function to counteract the low-dose TG-induced Ca^2+^ release response. As noted above, our protein expression experiments suggest that overexpression of the SERCA 3 pump isoform was easier to achieve compared to SERCA 2b and that the sum total of SERCA pump protein was greater in SERCA 3_OE_ Jurkat lymphocytes (Figure 1A). These results also suggest compensation mechanisms whereby changing expression levels of the SERCA 3 pump may also increase SERCA 2b pump expression, revealing a potential dynamic interdependence between expression levels of the two major pump isoforms. Indeed, this interpretation would explain the much-reduced Ca^2+^ release signal induced by low-dose TG in SERCA 3_OE_ cells compared to SERCA 2b_OE_ cells (Figure 1F). We observed the same effect manifest using low-dose tBHQ (1 μM, Figure 1G) as the Ca^2+^ release agonist on SERCA 2b_OE_ and SERCA 3_OE_ Jurkat lymphocytes, in which SERCA 3_OE_ cells reveal significantly reduced Ca^2+^ release responses compared to SERCA 2b_OE_ and control cells (ΔF 0.29 ± 0.02 and Ca^2+^ release rate ΔF/sec 1.61 × 10^−3^ for SERCA2b_OE_ & ΔF 0.20 ± 0.04 and Ca^2+^ release rate ΔF/sec 1.11 × 10^−3^ for SERCA3_OE_ vs. ΔF 0.28 ± 0.03 and Ca^2+^ release rate ΔF/sec 1.55 × 10^−3^ for control cells n = 3, *p* < 0.05). As above, overexpression of the SERCA 3 Ca^2+^ pump results in greater Ca^2+^-ATPase sequestering efficacy, particularly to low tBHQ concentrations that allow preferential targeting of the SERCA blocker to the SERCA 3 pumps. Indeed, tBHQ-induced responses in SERCA 2b_OE_ cells, which, as shown above, do not significantly change SERCA 3 expression levels, display responses similar to control lymphocytes, consistent with the interpretation that the SERCA 2b-regulated Ca^2+^ stores are less sensitive to tBHQ at low concentrations (Figure 1G).

We next investigated the effects of SERCA pump overexpression on Ca^2+^ signals induced by T cell receptor (TCR) activation using phytohemagglutinin A (PHA) application as a surrogate for TCR activation and inositol 1,4,5-trisphosphate (IP3) production [10]. Similar to the low-dose TG experiment, we observed that overexpression of both SERCA 2b and SERCA 3 Ca^2+^ pumps diminished TCR-activated Ca^2+^ release compared to control sham-transfected Jurkat lymphocytes (Figure 1H, SERCA 2b_OE_: ΔF 0.13 ± 0.008 and Ca^2+^ release rate ΔF/sec 4.74 × 10^−4^, n = 3, *p* < 0.05; SERCA 3_OE_: ΔF 0.17 ± 0.02 and Ca^2+^ release rate ΔF/sec 6.20 × 10^−4^, n = 3, *p* < 0.05 vs. control ΔF 0.26 ± 0.04 and Ca^2+^ release rate ΔF/sec 9.5 × 10^−4^). These experiments suggest that increasing expression/activity of SERCA pumps can exert a real-time suppressive effect on TCR/IP3-induced Ca^2+^ release from the endoplasmic reticulum (ER). Moreover, these constrained Ca^2+^ release responses in the presence of overexpressed SERCA pumps were also associated with reduced Ca^2+^ influx activation, presumably due to downregulation of the coupled store-depletion pathway (Figure 1H, SERCA 2b_OE_: ΔF 0.42 ± 0.015, n = 3, *p* < 0.05; SERCA 3_OE_: ΔF 0.41 ± 0.02, n = 3, *p* < 0.05 vs. control ΔF 0.46 ± 0.03, n = 3). These experiments with SERCA pump overexpression suggest that T lymphocytes can achieve significant reconfiguration of Ca^2+^ signaling patterns even with modest changes in SERCA expression levels, motivating further interest in SERCA ER Ca^2+^ pumps as a novel regulatory site for T cell management of antigen-activated signaling pathways.

### 2.2. Specific Knockout of SERCA 2b and SERCA 3 Ca^2+^-ATPases Further Clarifies T Lymphocyte Ca^2+^ Store Signaling Regulation at the Level of Ca^2+^ Release and Ca^2+^ Influx Pathways

To complement the overexpression studies and to further investigate the roles of SERCA pumps on T lymphocyte Ca^2+^ signaling pathways, we conducted experiments using SERCA 2b/SERCA 3 knockout Jurkat lymphocytes, testing responses to the same Ca^2+^ mobilizing agonists used in the overexpression studies.

**Figure 2:**
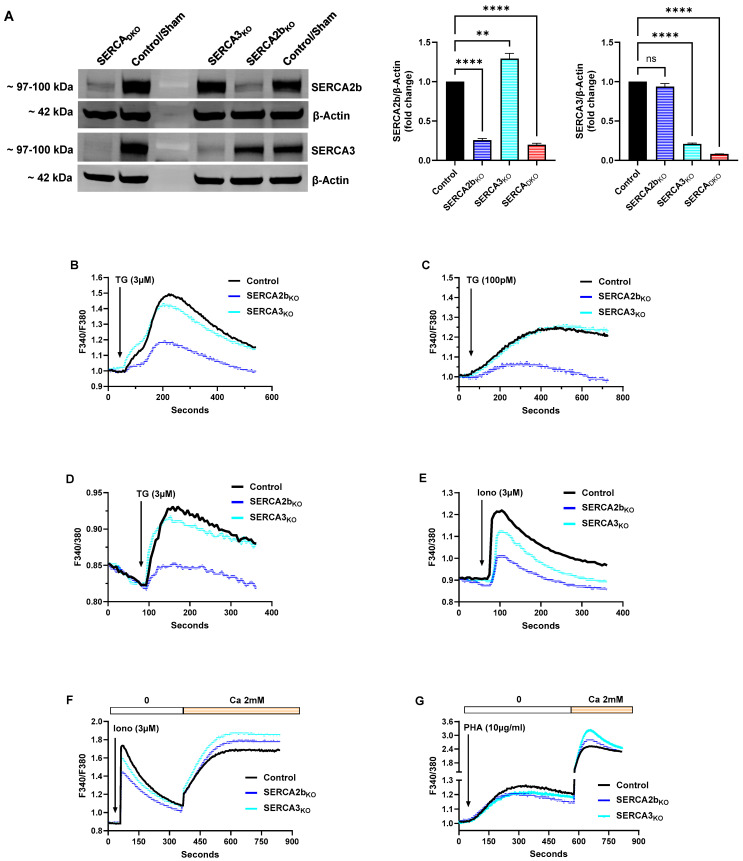
CRISPR-Based Knockout of SERCA Isoforms Significantly Alters Jurkat T Lymphocyte Ca^2+^ Store Status, Revealing Heightened Ca^2+^ Influx Coupling. (**A**) Representative Western blot image of CRISPR-mediated knockout (KO) of SERCA 2b, SERCA 3 and SERCA 2b/SERCA 3 double-knockout (DKO) experiments in Jurkat lymphocytes (see Section 4). Figure shows the approximate molecular weight of the SERCA proteins and β-actin protein bands as control. Additionally shown are the densitometry bar plots derived from band density quantification for SERCA 2b KO (dark blue bars), SERCA 3 KO (light blue bars) and SERCA 2b/SERCA 3 DKO (red bars) compared to control sham transfected cells (black bars). (**B**,**C**), Jurkat T lymphocytes were loaded with Fura-2 and suspended in Ca^2+^-free media (balanced salt solution plus 2 mM EGTA) in cuvettes for use in the large cell-number configuration (1 × 10^6^ cells/mL). (**B**) Jurkat cell Ca^2+^ release responses in SERCA 2b_KO_, SERCA 3_KO_ and control cells induced by the addition of TG (3 μM, arrow), as determined by the ratio of fluorescence changes at 340 and 380 nm (F340/380). (**C**) the same experiment as in *B* but with the application of low-dose TG (100 pM, arrow). (**D**,**E**) Ca^2+^ measurements were acquired from Jurkat T lymphocytes in the small cell-number configuration (30–60 cells per field) using microscope photometry (Section 4). (**D**) Jurkat cell Ca^2+^ release responses in SERCA 2b_KO_, SERCA 3_KO_ and control cells induced by the addition of TG (3 μM, arrow). (**E**) the same experiment as in (**D**) but with the application of ionomycin (3 μM, arrow). (**F**,**G**) Jurkat T lymphocytes were loaded with Fura-2 and suspended in Ca^2+^-free media (balanced salt solution plus 2 mM EGTA) in cuvettes for use in the large-cell number configuration (approximately 1 × 10^6^ cells/mL). (**F**) Jurkat cell Ca^2+^ release and influx responses in SERCA 2b_KO_, SERCA 3_KO_ and control cells induced by the addition of ionomycin (3 μM, arrow); Ca^2+^ influx responses were determined by the addition of Ca^2+^ (2 mM) to the cells suspended in cuvettes as indicated. (**G**) the same experiment as in (**F**) but with the application of PHA (10 μg/mL, arrow). Fluorescence traces of Ca^2+^ experiments shown are representative of three to five separate experiments with significant differences assessed via use of student *t* test. One-way ANOVA followed by Tukey’s multiple comparison tests were used to analyze Western blot data for SERCA expression levels with n = 3 (Section 4). Asterisks denote statistical significance with ** *p* < 0.005, **** *p* < 0.0001 and *ns* is not significant *p* > 0.05.

Figure 2A shows the Western blot results and the corresponding bar plots of band densitometry depicting specific knockout of SERCA 2b (SERCA2b_KO_), SERCA 3 (SERCA3_KO_) and double knockout of both pump isoforms (SERCA_DKO_). To achieve the specific knockout of SERCA pump isoforms, Jurkat lymphocytes were subjected to CRISPR gene editing methods in which cells were transfected using electroporation of specific nucleotide sequences for SERCA 2b (ATP2A2) and SERCA 3 (ATP2A3). Gene transfection efficiencies were quite high (85–95%), assessed as described for the overexpression experiments (see Section 4), and the protein expression levels were determined 5 days following transfection, and they revealed that we achieved significant reduction in SERCA 2b expression levels (Figure 2A: SERCA 2b_KO_ 15–20% of control; SERCA_DKO_ 15–20% of control) and SERCA 3 expression levels (Figure 2A: SERCA 3_KO_ 15–20% of control; SERCA_DKO_ 5–10% of control). Interestingly, similar to our SERCA overexpression experiments, we observed an apparent interdependence of SERCA 2b expression on changes in SERCA 3 levels, given we found that SERCA 2b pump expression is enhanced in SERCA 3_KO_ cells (Figure 2A). Indeed, previous studies have shown a similar effect during T cell activation, reporting reduced SERCA 3 Ca^2+^ pump expression accompanied by increases in expression of the SERCA 2b isoform [20,21,22]. We tested SERCA 2b_KO_ and SERCA 3_KO_ and SERCA_DKO_ Jurkat lymphocytes 5 days following transfection using the same protocols and Ca^2+^ activating agents as described above for the overexpression experiments.

Figure 2B shows a significant reduction in high-dose TG- (3 μM) induced Ca^2+^ release in SERCA 2b_KO_ T lymphocytes displaying the knockout phenotype (ΔF 0.19 ± 0.02 and Ca^2+^ release rate ΔF/sec 1.25 × 10^−3^, 38% of control Ca^2+^ release rate ΔF/sec 3.30 × 10^−3^, n = 5, *p* < 0.05). We observed a much smaller reduction in TG-induced Ca^2+^ release in SERCA 3_KO_ cells (Ca^2+^ release rate ΔF/sec 2.93 × 10^−3^, 88% of control). These results are consistent with previous studies indicating that TG primarily releases Ca^2+^ from the IP3-sensitive SERCA 2b-regulated store [23,24]. The small response still observed in SERCA 2b_KO_ lymphocytes under the knockout condition may be due to Ca^2+^ release primarily from SERCA 3-regulated Ca^2+^ stores, given that at these high TG concentrations, all SERCA pump isoforms will be inhibited. This interpretation is further supported by the results shown in Figure 2C with the application of the low-dose TG (100 pM) treatment. Reducing TG levels to these low concentrations clarifies substantially the specificity of TG’s targeted actions on the SERCA 2b Ca^2+^ pool, as we observed near complete abrogation of Ca^2+^ release activity in the SERCA 2b_KO_ cells (Figure 2C, 25% of control sham transfected cells, n = 3, *p* < 0.05), whereas virtually no change was observed in the SERCA 3_KO_ cells.

Similar to the SERCA overexpression studies, we performed additional experiments using small cell-number population samples to provide assurances of signal fidelity and response interpretations obtained with the standard large cell-number population measurements. Figure 2D,E show Ca^2+^ release patterns obtained using microscope photometry in SERCA 2b_KO_ and SERCA 3_KO_ Jurkat lymphocytes treated with high-dose Ca^2+^ release agonists TG (3 μM) and ionomycin (3 μM). These experiments are designed to aggressively empty the composite SERCA replenished Ca^2+^ stores with high dose TG and also assess the broader global increment of Ca^2+^ pools including TG-insensitive Ca^2+^ storage compartments using high dose ionomycin. Figure 2D shows a similar result to Figure 2B, in which the SERCA 2b_KO_ condition largely abolishes high-dose TG-induced Ca^2+^ release, while this response is mostly preserved in SERCA 3_KO_ T lymphocytes, suggesting, as before, greater selectivity of TG’s actions on the SERCA 2b Ca^2+^ store.

T cells expressing reduced SERCA 2b/SERCA 3 Ca^2+^-ATPases revealed an intriguing differential pattern in our small cell-number population experiments treated with high-dose ionomycin. We observed that both SERCA 2b_KO_ and SERCA 3_KO_ corresponded to a much-reduced ionomycin-induced Ca^2+^ release response indicating that reduced SERCA pump expression resulted in a more global state of depleted Ca^2+^ stores (Figure 2E). We noted, however, that SERCA 2b_KO_ appeared to reduce the composite Ca^2+^ pools to a significantly greater extent than SERCA 3_KO_ lymphocytes (Figure 2E, SERCA 2b_KO_: Ca^2+^ release rate ΔF/sec 3.20 × 10^−3^, 43% of control Ca^2+^ release rate ΔF/sec 7.40 × 10^−3^, *p* < 0.05; SERCA 3_KO_: Ca^2+^ release rate ΔF/sec 5.40 × 10^−3^, 73% of control, *p* < 0.05, n = 3). As mentioned previously, this effect may reveal a differential dynamic balance between distinct Ca^2+^ pools in T lymphocytes, suggesting that Ca^2+^ sequestered in SERCA 2b-regulated stores translocates or exchanges more readily than Ca^2+^ contained in the SERCA 3-regulated stores, given the greater loss of ionomycin-induced releasable Ca^2+^ in SERCA 2b_KO_ T cells. As mentioned previously, this effect may also be due in part to our observations that SERCA 2b_KO_ cells experience no significant compensatory increase in SERCA 3 expression, in contrast to the SERCA 3_KO_ cells, and thus have reduced total SERCA expression/function resulting in reduced Ca^2+^ storage, as revealed by high dose ionomycin treatment (Figure 2E).

Indeed, we did observe a greater sensitivity of T lymphocyte Ca^2+^ influx activation in cells with reduced SERCA 2b/SERCA 3 expression, consistent with reduced SERCA function, producing a leakier state of Ca^2+^ stores, as shown in Figure 2F. Thus, even though high-dose ionophore stimulated diminished Ca^2+^ release in SERCA 2b_KO_ and SERCA 3_KO_ T lymphocytes, we still observed larger Ca^2+^ influx responses when Ca^2+^ was added back to the cells (Figure 2F). Like the SERCA overexpression studies, these experiments suggest that T lymphocytes may be able to modulate coupling strength to the essential Ca^2+^ influx pathways via modest alterations in SERCA pump expression or activity.

We observed that a corresponding reduced SERCA expression could also alter T lymphocyte Ca^2+^ signals induced by TCR/IP3 activation using PHA treatment as a nonspecific TCR activator [10]. Like the high-dose TG and ionomycin treated cells, we observed diminished PHA-induced Ca^2+^ release responses in SERCA 2b_KO_ (Ca^2+^ release rate ΔF/sec 7.6 × 10^−4^, 76% of control Ca^2+^ release rate ΔF/sec 1.0 × 10^−3^, n = 3) and SERCA 3_KO_ (Ca^2+^ release rate ΔF/sec 7.6 × 10^−4^, 76% of control Ca^2+^ release rate ΔF/sec 1.0 × 10^−3^). Jurkat lymphocytes (Figure 2G). Reduced SERCA function thus diminishes IP3-mobilizable Ca^2+^ pools with a corresponding relatively more depleted Ca^2+^ store, as revealed by the significantly larger Ca^2+^ influx responses compared to sham-transfected Jurkat lymphocytes (Figure 2G, SERCA2b_KO_: ΔF 1.40 ± 0.07, SERCA3_KO_: ΔF 2.04 ± 0.09, n = 3, vs. control ΔF 1.32 ± 0.05, *p* < 0.05). We noted an intriguing distinction in Ca^2+^ influx responses with an apparent stronger activation of these pathways in SERCA 3_KO_ T cells in both ionomycin and PHA treated cells (Figure 2F,G), suggesting that SERCA 3-regulated Ca^2+^ pools exhibit incrementally more robust coupling to influx machinery than SERCA 2b-regulated pools. Thus, it appears that reduced SERCA 3 expression in the SERCA 3_KO_ cells results in the depletion of SERCA 3-regulated Ca^2+^ stores, along with measurably greater activation of Ca^2+^ influx pathways compared to SERCA 2b_KO_ cells containing reduced SERCA 2b pump expression.

We concluded our investigation of Ca^2+^ signal profiles in SERCA-expression-altered T lymphocytes by employing aggressive perturbation of ER Ca^2+^ stores via generation of a SERCA 2b/SERCA 3 double-knockout condition (SERCA_DKO_, Figure 2A). SERCA_DKO_ Jurkat lymphocytes were maintained in regular continuous culture conditions and presented as generally stable cells, albeit with reduced viability levels (see below). We tested the SERCA_DKO_ cells using the same battery of Ca^2+^ mobilizing agonists described in the foregoing experiments: high-dose TG and ionomycin, low-dose TG and tBHQ and PHA.

**Figure 3:**
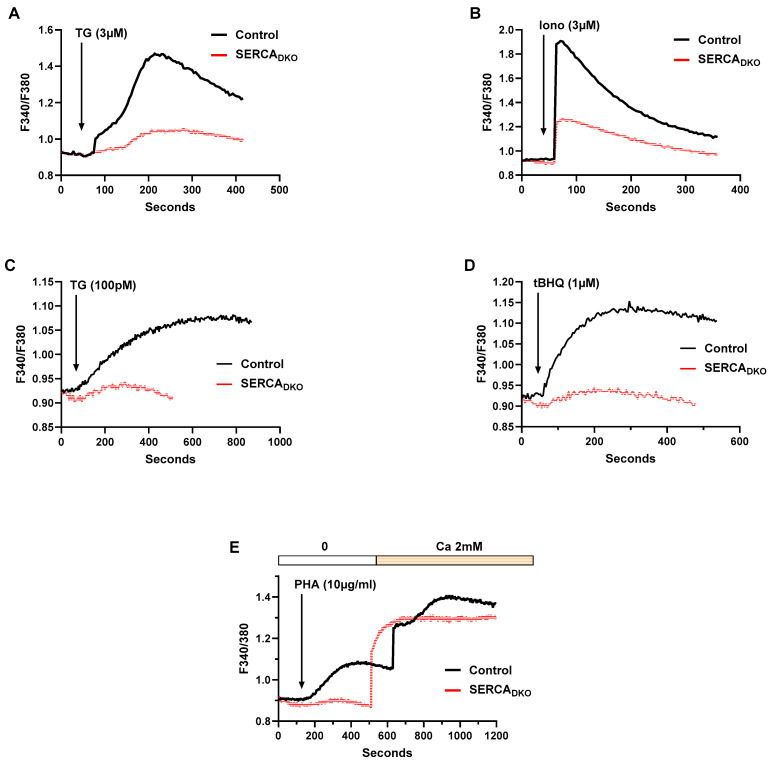
CRISPR-Mediated Double-Knockout of Jurkat Lymphocyte SERCA Pumps Severely Perturbs Ca^2+^ Stores and Further Clarifies Ca^2+^ Pool Agonist Sensitivity. (**A**–**E**) Jurkat T lymphocytes were loaded with Fura-2 and suspended in Ca^2+^-free media (balanced salt solution plus 2 mM EGTA) in cuvettes for use in the large cell-number configuration (approximately 1 × 10^6^ cells/mL). (**A**) Jurkat cell Ca^2+^ release responses in SERCA_DKO_ and control cells induced by the addition of TG (3 μM, arrow), as determined by the ratio of fluorescence changes at 340 and 380 nm (F340/380). (**B**) the same experiment as in (**A**) but with the application of ionomycin (3 μM, arrow). (**C**,**D**) Jurkat T lymphocytes were challenged with the low-dose SERCA blocker regimen. *C*, Jurkat cell Ca^2+^ release responses in SERCA_DKO_ and control cells induced by the addition of low-dose TG (100 pM, arrow), as determined by the ratio of fluorescence changes at 340 and 380 nm (F340/380). (**D**) the same experiment as in (**C**) but with the application of low-dose tBHQ (1 μM, arrow). (**E**) Jurkat cell Ca^2+^ release and influx responses in SERCA_DKO_ and control cells induced by the addition of PHA (10 μg/mL, arrow); Ca^2+^ influx responses were determined by the addition of Ca^2+^ (2 mM) to the cuvettes as indicated. Fluorescence traces of Ca^2+^ experiments shown are representative of three to six separate experiments with significant differences assessed via use of student *t* test.

Figure 3A,B show the Jurkat lymphocyte responses to high-dose TG and ionomycin, revealing near total abrogation of TG (Ca^2+^ release rate ΔF/sec 8.12 × 10^−4^, 23% of control Ca^2+^ release rate ΔF/sec 3.5 × 10^−3^, n = 3, Figure 3A) and ionophore (Ca^2+^ release rate ΔF/sec 1.24 × 10^−2^, 37% of control Ca^2+^ release rate ΔF/sec 3.34 × 10^−2^, n = 3, Figure 3B) -releasable Ca^2+^ pools in the SERCA_DKO_ cells. The small Ca^2+^ release observed in the high-dose TG experiment (Figure 3A) may be due to some residual SERCA pump expression, as complete knockout of SERCA expression is not achieved using these CRISPR-based methods (Figure 2A). The small increment of Ca^2+^ release with high-dose ionomycin may reflect Ca^2+^ release from non-SERCA regulated pools, yet clearly demonstrating that in the absence of robust SERCA function, this storage compartment is considerably depleted (Figure 3B).

Similarly, SERCA_DKO_ Jurkat T lymphocytes display near complete eradication of Ca^2+^ release responses to application of low-dose TG (100 pM, Ca^2+^ release rate ΔF/sec 1.15 × 10^−4^, 25% of control Ca^2+^ release rate ΔF/sec 4.62 × 10^−4^, n = 3, Figure 3C) and low-dose tBHQ (1 μM, Ca^2+^ release rate ΔF/sec 2.22 × 10^−4^, 16% of control Ca^2+^ release rate ΔF/sec 1.41 × 10^−3^, n = 3, Figure 3D). As mentioned previously, these are quite weak agonists at these concentrations, likely producing modest SERCA pump perturbations, and thus, it is not surprising that these responses have been largely abolished in the SERCA double knockout phenotype. Yet, SERCA_DKO_ T lymphocytes should represent a severe disruption of Ca^2+^ stores, a greater ER Ca^2+^ store perturbation than we observe in the single selective SERCA knockout experiments, in which some compensation or preservation of internal Ca^2+^ pools is likely mediated by residual SERCA pump function. Indeed, as shown in Figure 3E, we observed that PHA-induced TCR/IP3 activation failed to induce Ca^2+^ release in the SERCA_DKO_ lymphocytes, underscoring the severely depleted state of the Ca^2+^ stores. Thus, in this background of aggressively depleted Ca^2+^ stores, we observed high sensitivity and strong coupling to the Ca^2+^ influx pathway in SERCA_DKO_ T lymphocytes when Ca^2+^ levels (2 mM) were restored following TCR activation (Figure 3E, SERCA_DKO_: ΔF 0.43 ± 0.04 vs. sham-transfected control cells: ΔF 0.35 ± 0.03, n = 3, *p* < 0.05).

### 2.3. Pharmacologic SERCA Modulators and Altered SERCA Expression Levels Suggest Broad Integrated SERCA Regulation of Essential T Lymphocyte Functions

We shifted our investigation from SERCA effects on Ca^2+^ signaling pathways to a key subset of T lymphocyte functions including cell proliferation, cytokine secretion, glucose acquisition and oxidative stress management.

**Figure 4:**
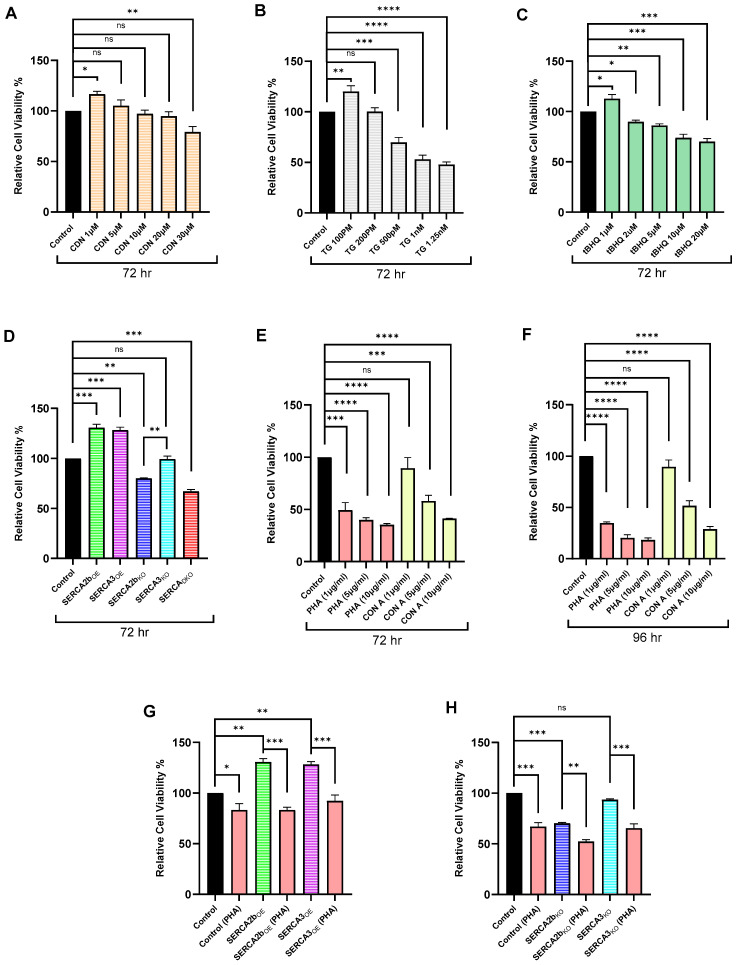
SERCA Modulators and Altered SERCA Expression Levels Exert Sensitive Regulatory Influence on Jurkat T Lymphocyte Growth Responses. For (**A**–**C**), Jurkat T lymphocytes were grown in the presence of the indicated concentrations of SERCA modulators for 72 h to determine levels of cell proliferation (Section 4). (**A**), Effects on cell growth for cells incubated with the indicated concentrations of CDN1163 (beige bars) relative to untreated controls (black bar). (**B**), Effects on cell growth for cells incubated with the indicated concentrations of TG (purple bars) relative to untreated controls (black bar). (**C**), Effects on cell growth for cells incubated with the indicated concentrations of tBHQ (green bars) relative to untreated controls (black bar). (**D**), Effects of SERCA overexpression, SERCA 2b_OE_ (green bar) and SERCA 3_OE_ (purple bar) on Jurkat T lymphocyte growth responses relative to untreated controls (black bar). Additionally depicted are the effects of SERCA knockout, SERCA 2b_KO_ (dark blue bar), SERCA 3_KO_ (light blue bar) and SERCA_DKO_ (red bar) on Jurkat T lymphocyte growth responses relative to untreated controls (black bar). For (**E**,**F**), Jurkat cell growth responses were determined in the presence of T cell mitogens PHA and Con A for the indicated time intervals. (**E**), Effects on cell growth for cells incubated with the indicated concentrations of PHA (pink bars) and Con A (yellow bars) relative to untreated controls (black bar) for 72 h. (**F**), Effects on cell growth for cells incubated with the indicated concentrations of PHA (pink bars) and Con A (yellow bars) relative to untreated controls (black bar) for 96 h. For (**G**,**H**), Jurkat T lymphocyte growth responses to PHA stimulation were measured in cells with altered SERCA expression levels. *G*, Effects of SERCA 2b_OE_ on PHA-stimulated (pink bar) Jurkat lymphocyte growth responses compared to unstimulated SERCA 2b_OE_ (green bar) and sham transfected PHA-stimulated (pink bar) and unstimulated controls (black bar). Additionally depicted are the effects of SERCA 3_OE_ on PHA-stimulated (pink bar) Jurkat lymphocyte growth responses compared to unstimulated SERCA 3_OE_ (purple bar) and sham transfected PHA-stimulated (pink bar) and unstimulated controls (black bar). (**H**), Effects of SERCA 2b_KO_ on PHA-stimulated (pink bar) Jurkat lymphocyte growth responses compared to unstimulated SERCA 2b_KO_ (dark blue bar) and sham transfected PHA-stimulated (pink bar) and unstimulated controls (black bar). Additionally depicted are the Effects of SERCA 3_KO_ on PHA-stimulated (pink bar) Jurkat lymphocyte growth responses compared to unstimulated SERCA 3_KO_ (light blue bar) and sham transfected PHA-stimulated (pink bar) and unstimulated controls (black bar). Data are expressed as the mean ± SD, n = 3 replicates. One-way ANOVA followed by Dunnett’s multiple comparison test was used to calculate *p* values (**A**–**F**), except for (**D**), where Tukey’s multiple comparisons test were used. Two-way ANOVA employing Tukey’s multiple comparisons test was used to calculate *p* values (**G**–**H**). Asterisks denote statistical significance with * *p* < 0.05, ** *p* < 0.005, *** *p* < 0.001, **** *p* < 0.0001, and *ns* is not significant *p* > 0.05.

Figure 4 summarizes Jurkat lymphocyte proliferation responses to various experimental treatments that alter SERCA function or expression levels. Figure 4A shows dose response effects of treating T lymphocytes with increasing concentrations of the SERCA activator CDN1163 for 72 h. We have previously reported that extended incubation (>24 h) with CDN1163 increased Ca^2+^ storage in the Jurkat lymphocyte SERCA-regulated Ca^2+^ pools, with a preferential effect on the SERCA 2b Ca^2+^ stores [19]. We found that incubation with CDN1163 concentrations in the low micromolar range produced a modest increase in lymphocyte proliferation, suggesting, as we previously noted, a salutary effect on T lymphocyte growth that correlated with enhanced Ca^2+^ storage capacity [19].

Figure 4B,C show the effects on Jurkat lymphocyte cell growth using the SERCA blockers TG and tBHQ. These experiments revealed the intriguing phenomenon in which low-dose SERCA inhibition with its associated ER Ca^2+^ increased permeability, and cytosolic Ca^2+^ elevation can stimulate T lymphocyte cell growth. Thus, with imposing moderate ER stress stimulus with TG levels at low concentrations (≤100 pM) and also with low tBHQ levels (≤1 μM), we observed measurable increases in cell proliferation. Indeed, TG was initially categorized as a tumor promoter due to its actions to block SERCA function and stimulate a growth-promoting Ca^2+^ signal [25]. However, increasing TG levels modestly above a sharp transition point (≥200 pM) produced, in contrast, a pronounced inhibition on T lymphocyte growth, suggesting more aggressive perturbation of SERCA-regulated Ca^2+^ stores (Figure 4B). We observed a similar effect for tBHQ exposure, with cell growth inhibition developing at concentrations greater than one micromolar (Figure 4C).

Perturbation of SERCA functions via altered expression levels also reveals interconnections between T lymphocyte growth control and SERCA-regulated Ca^2+^ store function. Figure 4D shows that overexpression of both SERCA 2b and SERCA 3 significantly boost Jurkat lymphocyte proliferation. This effect is consistent with our previous study [19] and Figure 4A, in which pharmacologic SERCA activation with CDN1163 application contributes to increased cell proliferation associated with increased Ca^2+^ storage capacity. Indeed, this is also the result we reported in Figure 1B–E, demonstrating increased Ca^2+^ storage in SERCA 2b_OE_ and SERCA 3_OE_ T lymphocytes, as revealed by high dose TG and ionomycin treatment. We observed, moreover, a correspondingly diminished growth response in SERCA 2b_KO_ and SERCA_DKO_ cells (Figure 4D). These results hint at a parallel relationship with increased SERCA expression/activity producing augmentation of lymphocyte growth, whereas decreased SERCA expression/activity result in diminished lymphocyte growth. As mentioned above (Figure 2 and Figure 3), we do find that genetic knockout of SERCA 2b and SERCA 3 results in substantial reduction in ER Ca^2+^ storage capacity. These experiments further suggest SERCA isoform specificity in regulation of T lymphocyte growth responses. Figure 4D shows, for example, that specific knockout of the SERCA 2b pump significantly decreases cell growth, while knockout of the SERCA 3 pump does not. This result also aligns with our previous work, showing that CDN1163 appears to preferentially act on increasing the SERCA 2b Ca^2+^ pools, conferring increased growth and cellular resilience via specific action on the SERCA 2b isoform. Thus, our findings suggest differential modes of T lymphocyte signaling regulation exerted by the SERCA pumps, with SERCA 2b integrated more prominently in cell growth regulation compared to SERCA 3 Ca^2+^ pumps.

We next examined the effect of altered SERCA expression levels on T lymphocyte growth responses induced by canonical Ca^2+^ signaling pathways activated by the commonly employed lectin T cell mitogens PHA and Concanavalin A (Con A). Figure 4E,F show dose response effects on Jurkat lymphocyte growth responses for cells exposed to PHA and Con A for extended incubation periods (72 and 96 h). The experiments revealed pronounced and increasing growth suppression with exposure to the two mitogens over the 96 h incubation period. PHA is a well-known mimic of the antigen-activated TCR/IP3/Ca^2+^ pathway, which initially stimulates T lymphocyte proliferation followed by triggering of activation-induced cell death pathways and, ultimately, cell growth suppression [26,27]. Con A also triggers the activation of T cell Ca^2+^ pathways, albeit via recruitment of different signaling mediators [28]; but like PHA, Con A also induces growth suppression in T lymphocyte populations, though not to the same degree as observed with PHA in Jurkat lymphocytes (Figure 4E,F). Using this lectin treatment protocol, we tested whether increasing or decreasing SERCA expression levels could modulate the growth-suppressing action of PHA on Jurkat lymphocytes. As shown in Figure 4G,H, neither SERCA 2b/SERCA 3 overexpression nor SERCA 2b/SERCA 3 knockout could compensate for or protect T lymphocytes from the initial effects of PHA to reduce cell growth (24 h). Indeed, the increase in cell proliferation observed in SERCA 2b_OE_ and SERCA 3_OE_ Jurkat cells (Figure 4D) was eradicated in cells treated with PHA (10 μg/mL) for 24 h. Moreover, treating SERCA 2b_KO_ and SERCA 3_KO_ T lymphocytes with PHA (10 μg/mL***)*** caused an even greater reduction in cell proliferation than was observed in the knockout condition alone (Figure 4H). Thus, altering Ca^2+^ store levels and/or Ca^2+^ influx activity as we observed in SERCA 2b_OE_/SERCA 3_OE_ or SERCA 2b_KO_/SERCA 3_KO_ Jurkat lymphocytes (Figure 1 and Figure 2, respectively) was insufficient to reconfigure or abort growth-suppressing TCR signals unaccompanied by other essential signaling input that would normally emerge from valid antigen activation.

**Figure 5:**
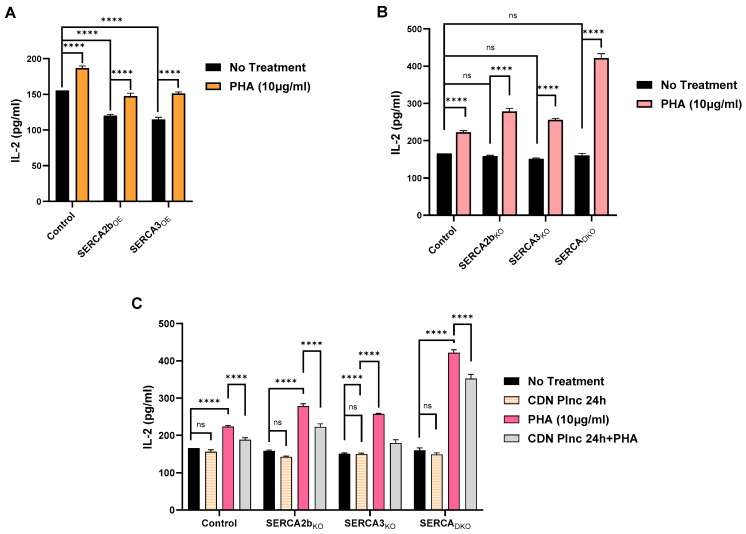
Altered SERCA Expression Levels Regulate Tonic and Stimulated Jurkat T Lymphocyte IL-2 Secretion. (**A**), Levels of IL-2 were measured in PHA-stimulated and unstimulated Jurkat T lymphocyte culture supernatants (Section 4). SERCA 2b_OE_ and SERCA 3_OE_ Jurkat cells were treated with PHA (10 μg/mL, yellow bars), and IL-2 levels were compared to unstimulated SERCA 2b_OE_ and SERCA 3_OE_ (black bars) cells. IL-2 levels from SERCA 2b_OE_ and SERCA 3_OE_ cells were also compared to untransfected control Jurkat lymphocytes from either the unstimulated (black bar) or PHA-stimulated (yellow bar) condition. (**B**), SERCA 2b_KO_, SERCA 3_KO_ and SERCA_DKO_ Jurkat cells were treated with PHA (10 μg/mL, pink bars), and IL-2 levels were compared to unstimulated SERCA 2b_KO_, SERCA 3_KO_ and SERCA_DKO_ (black bars) cells. IL-2 levels from SERCA 2b_KO_, SERCA_KO_ and SERCA_DKO_ cells were also compared to untransfected control Jurkat lymphocytes from either the unstimulated (black bar) or PHA-stimulated (pink bar) condition. (**C**), Similar to (**B**), measuring IL-2 levels in SERCA knockout Jurkat lymphocytes but with testing the effects of preincubation with CDN1163 (10 μM, 24 h) in both PHA-stimulated and unstimulated conditions. IL-2 levels were determined in SERCA 2b_KO_, SERCA 3_KO_ and SERCA_DKO_ cells stimulated with PHA (10 μg/mL, pink bars) and compared with levels observed in PHA-treated cells preincubated with CDN1163 (10 μM, 24 h, grey bars). The same experiment was conducted in the SERCA knockout cells with (*yellow bars*) or without (black bars) CDN1163 pretreatment. Levels of IL-2 in the SERCA knockout lymphocytes were also compared to the same experimental conditions performed on untransfected Jurkat lymphocytes, denoted as the control condition in the figure. Data are expressed as the mean ± SD, n = 3 replicates. Two-way ANOVA followed by Tukey’s multiple comparisons test was used to calculate *p* values. Asterisks denote statistical significance with **** *p* < 0.0001 and *ns* is not significant *p* > 0.05.

A key signaling output pathway in T lymphocyte function is the antigen stimulated production of cytokine factors. In our study, we examined the TCR activation pathway that results in IL-2 secretion, utilizing the commonly employed surrogate actions of PHA to nonspecifically stimulate the TCR/IP3/Ca^2+^ signal upstream of IL-2 synthesis. Figure 5A shows the effect of PHA (10 μg/mL) to induce IL-2 secretion above baseline untreated Jurkat lymphocytes. We noted that IL-2 secretion was depressed in SERCA 2b_OE_ and SERCA 3_OE_ cells and that PHA stimulation could increase IL-2 levels in the SERCA-overexpressed condition, albeit at levels still below control untreated T lymphocytes (Figure 5A). This result aligns with our observations in Figure 1H, showing that PHA treatment in SERCA-overexpressing Jurkat lymphocytes induces both reduced Ca^2+^ release and Ca^2+^ influx, which would explain reduced IL-2 secretion given this cytokine’s strong dependence on recruitment of the Ca^2+^ influx pathway [4,5]. In contrast to SERCA overexpression, SERCA knockout did not significantly affect IL-2 production (Figure 5B). Indeed, this difference may be attributable to the augmented Ca^2+^ influx activity we noted in Figure 2 in SERCA 2b_KO_ and SERCA 3_KO_ cells. This interpretation would also be consistent with our observations that PHA stimulation in the SERCA knockout phenotype is still capable of elevating IL-2 production (Figure 5B), even though Ca^2+^ release induced by PHA was curtailed presumably by reduced SERCA activity and reduced Ca^2+^ store levels (Figure 2G). Perhaps the most compelling validation of this idea is our finding that the largest increase we observed in PHA-induced IL-2 production was in the SERCA_DKO_ Jurkat lymphocyte (Figure 5B). This finding correlates well with our observations that the PHA-induced Ca^2+^ influx response was rapidly and robustly activated in the SERCA_DKO_ cells, even with nearly eradicated PHA-stimulated Ca^2+^ release compared to sham transfected Jurkat controls (Figure 3E).

We extended our experiments shown in Figure 5B to assess the effects of the pharmacological modulation of SERCA pumps using the pre-incubation regimen with the SERCA activator CDN1163 reported in our previous study [19]. Figure 5C reveals that CDN1163 pre-incubation (24 h) failed to significantly alter IL-2 production in unstimulated Jurkat lymphocytes. However, we observed a significant reduction in IL-2 secretion in PHA-stimulated T lymphocytes pre-treated with CDN1163 (Figure 5C). Thus, this result is analogous to the SERCA overexpression condition and presumably attributable to increased SERCA functional activity with a corresponding increase in Ca^2+^ store loading, thereby diminishing Ca^2+^ influx and IL-2 synthesis. Indeed, we even observed that CDN1163 exposure could significantly suppress the strong PHA-induced IL-2 synthesis in the Jurkat SERCA_DKO_ lymphocyte, underscoring the efficacy of pharmacological SERCA activation in modulating T cell activation pathways (Figure 5C).

**Figure 6:**
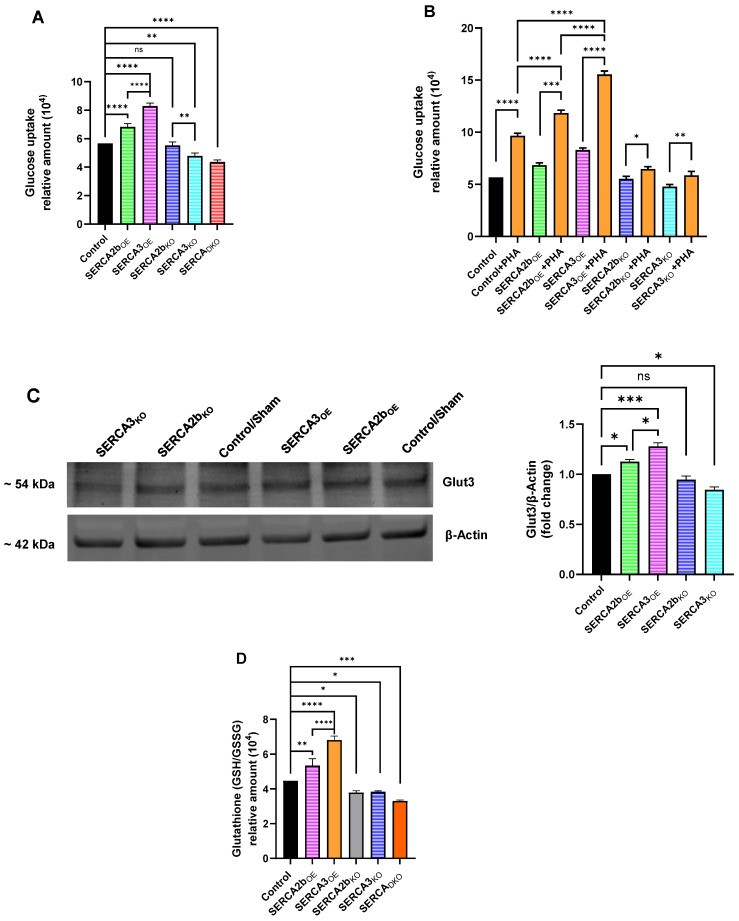
Altered Expression Levels Reveal SERCA Isoform-Specific Regulation on Jurkat T Lymphocyte Functional Parameters Spanning Glucose Uptake and Glutathione Antioxidant Production. (**A**), Glucose uptake was measured in Jurkat T lymphocytes with altered SERCA expression levels (Section 4). Glucose uptake in SERCA 2b_OE_ (green bar), SERCA 3_OE_ (purple bar), SERCA 2b_KO_ (dark blue bar), SERCA 3_KO_ (light blue bar) and SERCA_DKO_ (red bar) lymphocytes was measured and compared to untransfected control Jurkat lymphocytes (black bar). (**B**), similar to *A* but including PHA-stimulated effects on glucose uptake. Yellow bars denote glucose uptake, respectively, in SERCA 2b_OE_ (green bar), SERCA 3_OE_ (purple bar), SERCA 2b_KO_ (dark blue bar), SERCA 3_KO_ (light blue bar), SERCA_DKO_ (red bar) and control unstransfected (black bar) PHA-stimulated Jurkat lymphocytes. (**C**), Representative Western blot image of expression levels of the Glucose Transporter 3 (Glut3) protein in SERCA 2b_OE_, SERCA 3_OE_, SERCA 2b_KO_ and SERCA 3_KO_ Jurkat T lymphocytes. Figure shows the approximate molecular weight of the Glut3 protein and β-actin protein bands as control. Additionally shown are the densitometry bar plots derived from band density quantification for SERCA 2b_OE_ (green bar), SERCA 3_OE_ (purple bar), SERCA 2b_KO_ (dark blue bar) and SERCA 3_KO_ (light blue bar) cells compared to control sham transfected cells (black bars). (**D**), Glutathione (GSH/GSSG) levels were measured in Jurkat T lymphocytes with altered SERCA expression levels (Section 4). Glutathione levels in SERCA 2b_OE_ (purple bar), SERCA 3_OE_ (yellow bar), SERCA 2b_KO_ (grey bar), SERCA 3_KO_ (blue bar) and SERCA_DKO_ (orange bar) lymphocytes were measured and compared to untransfected control Jurkat lymphocytes (black bar). Data are expressed as the mean ± SD, n = 3 replicates. One-way ANOVA followed by Tukey’s multiple comparison test were used to calculate *p* values (**A**,**C**,**D**). Two-way ANOVA employing Tukey’s multiple comparisons test were used to calculate *p* values (**B**). Asterisks denote statistical significance with * *p* < 0.05, ** *p* < 0.005, *** *p* < 0.001, **** *p* < 0.0001 and *ns* is not significant *p* > 0.05.

Antigen-activated T lymphocytes enter complex and protracted phases of cell proliferation and differentiation and therefore exhibit high demand for glucose uptake and utilization [29,30]. We were interested in examining the effect of altered SERCA pump expression on glucose uptake activity in Jurkat lymphocytes, given the demands on activated proliferating cells and also recognizing that SERCA pumps themselves are significant energy-consuming ion transporters operating continuously in the environment of a Ca^2+^ permeable ER membrane [31,32]. Figure 6A shows that overexpression of the SERCA pumps significantly increases glucose uptake in Jurkat transfected cells, presumably to accommodate in part the increased demand on ATP synthesis needed for enhanced active Ca^2+^ transport. Furthermore, intriguingly, just as in our experiments testing cell proliferation and Ca^2+^ signaling responses, we observed a SERCA isoform difference in glucose uptake measurements with SERCA 3_OE_ cells driving significantly greater glucose uptake than was observed in the SERCA 2b_OE_ T lymphocytes. In keeping with this observation, we noted a significant decrease in glucose uptake in SERCA 3_KO_ T lymphocytes compared to the SERCA 2b_KO_ cells (Figure 6A), further suggesting a greater dependence on glucose utilization for the SERCA 3-regulated Ca^2+^ stores. Stimulation of the TCR/IP3/Ca^2+^ pathway with PHA treatment also increased glucose uptake compared to untreated Jurkat lymphocytes and was associated with a still greater increment of glucose uptake in SERCA 2b_OE_ and SERCA 3_OE_ cells with, yet again, a greater increase observed in SERCA 3_OE_ Jurkat cells relative to SERCA 2b_OE_ cells (Figure 6B). PHA-induced increases in glucose uptake were still measurable in SERCA 2b_KO_ and SERCA 3_KO_ T lymphocytes, albeit at much-reduced levels compared to the SERCA-overexpression condition (Figure 6B). This result may reflect reduced energy demands in a reduced Ca^2+^ storage/SERCA-expressing environment. Indeed, part of the enhanced sensitivity of glucose uptake we observed in SERCA 3_OE_ and SERCA 3_KO_ T lymphocytes may derive from a uniquely heightened linkage to expression of the Glut3 glucose transporter isoform [33]. T cells may use changes in SERCA 3 expression levels or activity as a sensor for energy utilization, coupling changes to SERCA 3-regulated Ca^2+^ store functions to increased/decreased transcription of the high-affinity Glut3 transporter to accommodate changing T cell energy demands. Figure 6C shows overexpression of the SERCA 3 pump results in significantly greater expression levels of Glut3 compared to SERCA 2b overexpression, and similarly, knockout of SERCA 3 correspondingly appears to reduce Glut3 transporter expression to a greater extent than SERCA 2b knockout Jurkat lymphocytes.

Our results suggest that SERCA expression levels can influence T lymphocyte energy homeostasis via glucose uptake pathways. We were thus motivated to investigate possible SERCA involvement in common T cell dysfunctional states related to oxidative stress, which are increasingly suspected contributors to maladaptive T cell signaling dynamics, including T cell disrupted function in the tumor microenvironment [34,35]. Figure 6D shows that altered SERCA expression is associated with corresponding changes to the production of the common antioxidant mediator reduced glutathione (GSH). We again noted the same SERCA isoform difference as was observed in the glucose uptake experiments with SERCA 3_OE_ cells associated with significantly greater GSH production than was observed in the SERCA 2b_OE_ cells (Figure 6D). Multiple parameters in our study thus suggest differential roles in T cell biology for the SERCA 2b and SERCA 3 Ca^2+^ pumps. Figure 6D further implicates SERCA regulation of GSH antioxidant production as we observed significant reduction in GSH levels in SERCA 2b_KO_, SERCA 3_KO_ and SERCA_DKO_ Jurkat lymphocytes.

## 3. Discussion

The SERCA Ca^2+^ pumps occupy a strategic position in cellular homeostasis in both regulating key Ca^2+^ signal-activating events as well as greatly influencing major ER organellar functions via their actions to manage essential Ca^2+^ levels in the ER [32]. Much of the previous work has advanced our understanding of the multi-functional roles of the SERCA pumps, perhaps most conspicuously in muscle cells where they are expressed at high levels and operate in a uniquely differentiated environment [36,37]. In contrast, much less is known about complex integrated SERCA functions in T lymphocyte signaling and cellular homeostasis. Our objective in this study was to begin an assessment of the effects of changing SERCA expression levels on key T cell functions. For this investigation, we used the commonly employed Jurkat T cell line owing to significant advantages provided by its clonal homogeneity, ease of genetic manipulation and extensive characterization. Indeed, Jurkat lymphocytes offer a good functional approximation to primary T lymphocytes, particularly as it relates to the TCR-activated Ca^2+^ pathway [9,10,11,12]. Moreover, our previous study reported a significant overlap in the pharmacological regulation of SERCA function in rat primary spleen lymphocytes and Jurkat lymphocytes, adding further support for their use in SERCA studies in T lymphocytes [19].

We find that with even modest SERCA overexpression, we can produce a T cell phenotype exhibiting measurable increases in Ca^2+^ storage capacity. This observation was supported in our experiments, showing that both global SERCA blockade with high TG concentrations and global Ca^2+^ store discharge with high ionomycin concentrations revealed augmented Ca^2+^ store levels when both SERCA 2b and SERCA 3 were overexpressed. Thus, T lymphocytes can clearly rapidly modulate Ca^2+^ levels stored in the ER by even limited increases in SERCA expression and/or function, allowing for shaping tailored Ca^2+^ signal patterns ostensibly linked to antigen stimulus strength.

We find, moreover, when applying more scaled targeted SERCA perturbation with low TG/tBHQ doses that upregulation of SERCA expression readily compensates for the SERCA-blocker-induced ER leak resulting in contained Ca^2+^ release and, presumably, preservation of ER Ca^2+^ store integrity. This result underscores the potential value of enhanced SERCA function in disease states wherein ER Ca^2+^ store and SERCA function appear to be compromised, including immune dysfunction, diabetes and neurodegeneration [38]. This dynamic action of increased SERCA pump expression also translated to the more physiological TCR signaling pathway, given we find that overexpression of both SERCA 2b and SERCA 3 significantly suppresses ER Ca^2+^ release and the corresponding activation of Ca^2+^ influx in cells stimulated with the TCR agonist PHA. This result again suggests that rapid recruitment of SERCA pump activation can be integrated into the complex Ca^2+^ signaling patterns intricately adapted to mediate specialized T cell functions coupled to TCR signaling output.

Given the unique metabolic demands proliferating and differentiated T lymphocytes encounter, we were motivated to perform companion studies to the SERCA overexpression experiments by introducing SERCA pump perturbations attributable to reduced SERCA expression levels. We have thus significantly reduced expression of the SERCA pumps using our CRISPR gene knockout approaches in Jurkat lymphocytes, producing SERCA 2b and SERCA 3 selective knockout, as well as SERCA 2b/SERCA 3 double knockout cell populations. Jurkat lymphocytes carrying the SERCA knockout phenotypes further clarified relationships among the SERCA 2b and SERCA 3 Ca^2+^ regulated stores, as we noted in our previous work, identifying significant selectivity of the low dose TG stimulus for SERCA 2b-regulated Ca^2+^ stores and, similarly, selectivity of the low dose tBHQ stimulus for SERCA 3-regulated Ca^2+^ pools [19]. Not surprisingly, genetic knockout of the SERCA pumps consistently resulted in T lymphocytes exhibiting reduced Ca^2+^ release responses to all Ca^2+^ mobilizing agonists. Thus, the SERCA knockout condition appeared to produce a T lymphocyte with diminished capacity to load leakier ER Ca^2+^ stores that clearly associated with increased Ca^2+^ influx activation, most likely due to a stronger depletion-activated signal in cells expressing reduced SERCA pump function. This was demonstrated most clearly in T lymphocytes with the severest ER perturbation in the SERCA 2b/SERCA 3 double knockout condition challenged with PHA-induced TCR activation. TCR activation with IP3 production resulted in nearly eradicated Ca^2+^ release in double knockout cells yet exhibited rapid and robust coupling to the Ca^2+^ influx pathway. Like the SERCA overexpression experiments, the SERCA knockout studies reveal fundamental mechanisms whereby T lymphocyte tonal SERCA function can directly influence recruitment of critical Ca^2+^ influx pathways via their ability to control the status of ER Ca^2+^ stores.

SERCA expression levels with corresponding changes in SERCA functional activity can directly downregulate or upregulate Ca^2+^ store properties, which are linked to critical T lymphocyte functions such as cell proliferation, cytokine synthesis/release and energy utilization. We thus extended our investigation to examine the effects of altering SERCA function, via changing expression levels or introducing SERCA pharmacological modulators, on these essential T lymphocyte functions. The general unifying pattern we observed in these experiments was that treatments that could be expected to increase SERCA function resulted in more robust cell proliferation. These observations highlight the potential strategic value of activating SERCA functions, either via genetic methods or pharmacologic agents, to increase cellular viability and resilience in immunopathologic states arising due to deficient T lymphocyte proliferation. Indeed, there is currently considerable interest in developing engineered T cell signaling elements that confer resilience and efficacy in promoting cytotoxic T cell function in the tumor microenvironment [13]. A recent study demonstrated improved T cell durability and efficacy in tumor control after introducing glucose transporter (Glut) overexpression, and, given our findings, a similar potentially profitable target to counter the tumor T cell exhaustion phenotype would be increased SERCA pump expression [30]. Indeed, similar to the Glut overexpression studies, our work also suggests multiple potential salutary effects of SERCA overexpression and/or functional upregulation, which include improved cell viability, glucose uptake and antioxidant protection.

In addition to reporting general SERCA functional regulation of T lymphocyte signaling regimes, we also find an intriguing panel of effects that suggest specifically distinct cellular functions ascribed to SERCA 2b and SERCA 3-regulated Ca^2+^ stores. We found, for example, a nonoverlapping expression profile of the SERCA 2b and SERCA 3 Ca^2+^ pumps; notably, increasing or decreasing SERCA 3 expression appears to exert linked changes to SERCA 2b expression levels, whereas the converse was not observed in that changes in SERCA 2b expression did not significantly alter SERCA 3 expression levels. Thus, there may be a built-in interdependent bias in the T lymphocyte SERCA system whereby changes to SERCA 3 function elicit compensatory changes to the SERCA 2b-regulated Ca^2+^ pools, which are generally believed to be the major TCR/IP3 targeted Ca^2+^ stores in T lymphocytes [2,23]. It may be, for example, that changes in SERCA 3 expression levels and corresponding alterations in SERCA 3-regulated Ca^2+^ stores serve as a type of sensor serving to recruit more SERCA 2b expression and function to augment SERCA 2b Ca^2+^ stores as an adaptive response to preserve ER organelle integrity. Indeed, this may be a response mechanism to confer protection or resilience to ER stress signals, metabolic or otherwise, similar to SERCA 3-mediated upregulation of glucose transporter activity and glucose uptake mentioned previously. This effect is consistent with our SERCA 3 overexpression experiments, in which we also observed a concurrent increase in SERCA 2b levels and thus a general overall increase in SERCA transporter protein levels, which appears to manifest in increased stored Ca^2+^ as well as rapidly blunted weak agonist-induced Ca^2+^ release, likely due to robust SERCA pump activity.

In addition to differences in SERCA protein expression patterns, we also noted a suite of biological effects that were different between the SERCA 2b and SERCA 3 pump isoforms. Knockout of SERCA 2b expression, for example, produced Jurkat lymphocytes with significantly reduced cell viability, a phenomenon we also observed in the SERCA 2b/3 double knockout cells. In contrast, SERCA 3 knockout T lymphocytes did not demonstrate significantly altered cell viability; and, moreover, SERCA 3 expression appeared to be more prominently linked to glucose utilization than SERCA 2b Ca^2+^ pumps, with SERCA 3 protein associated more sensitively to changes in glucose uptake activity, possibly via its ability to specifically regulate recruitment of the Glut3 glucose transporter. These preliminary insights reveal distinct integrated functions of the SERCA 2b and SERCA 3 pump isoforms within the broader landscape of T lymphocyte biology. SERCA 2b Ca^2+^ pumps may regulate Ca^2+^ pools integrally linked to cell activation/proliferation, whereas SERCA 3 regulated Ca^2+^ stores may be operating in more ancillary pathways that interface with energy and metabolic demands and the closely allied responses underlying regulation of ER and oxidative stress. Indeed, this explanation may explain why we also observe greater production of the global antioxidant reduced glutathione in SERCA 3 overexpressing Jurkat lymphocytes.

In this study, we have examined a spectrum of functional T cell parameters potentially regulated by SERCA pump activity; however, future studies emerge that can be undertaken to examine more closely particular effects of altered SERCA function on mitochondrial bioenergetics, given we observed SERCA-level regulation on glucose uptake and glucose transporter expression. Similarly, future experiments designed to further examine and differentiate potential roles of SERCA pumps in regulating the ER stress response, closely related to mitochondrial integrity and function, would also provide valuable new insight into SERCA-regulation of the broader T lymphocyte signaling landscape.

## 4. Materials and Methods

### 4.1. Materials

Fura 2/AM (fura 2 acetoxymethylester), pluronic acid, RPMI-160, fetal bovine se-rum (FBS), streptomycin and penicillin were obtained from Thermo Fisher Scientific (Waltham, MA, USA). Thapsigargin and cell/tissue culture flasks were obtained from Santa Cruz Biotechnology, Inc. (Dallas, TX, USA). Phytohemagglutinin (PHA) and 2,5-di-(tert butyl)-1,4-benzohydroquinone (tBHQ) were obtained from Sigma-Aldrich (St. Louis, MO, USA). Coverslips (35 mm Dish, No. 1.5 Coverslip, 14 mm Glass Diame-ter, High Adherence) were obtained from MatTek life Sciences (Ashland, MA, USA). CDN1163 was from Bio-Techne (Minneapolis, MN, USA).

### 4.2. Cell Culture

Jurkat cells (Clone E6–1, ATCC TIB-152) were maintained in RPMI-1640 medium supplemented with 10% fetal bovine serum, 2 mM L-glutamine, penicillin (100 IU/mL) and streptomycin (100 μg/mL) and grown at 37 °C in a humidified atmosphere (95% air, 5% CO_2_). Cells were maintained and expanded in either 25 cm^2^ (T25) or 75 cm^2^ (T75) tissue culture flasks. Cell density was not allowed to exceed 3 × 10^6^ cells/mL, and cultures were generally maintained at a cell concentration between 1 × 10^5^ and 1 × 10^6^ viable cells/mL. Fresh medium was added every 2 to 3 days, depending on cell density.

### 4.3. Calcium Large Cell-Number Assays

Cells (approximately 1 × 10^6^ cells/mL) were washed in Ca^2+^-containing (1.8 mM) Hanks balanced salt solution (HBSS) and loaded with 1.5 μM fura-2/AM in 20% (*w/v*) Pluronic F-127 and incubated for one hour at 37 °C. After loading, the cells were washed twice with HBSS and incubated at 37 °C for an additional 30 min to allow for de-esterification of the dye. Cells loaded with fura 2/AM were kept in the dark at room temperature throughout the experiments. Changes in cytosolic Ca^2+^ were measured in cell population experiments using a fluorescence spectrophotometer equipped with a thermostatically controlled sample compartment (PTI, Lawrenceville, NJ, USA), permitting continuous stirring of samples in the cuvette. All measurements were carried out at room temperature (25 °C). To achieve Ca^2+^-free conditions, EGTA (2 mM) was added to chelate extracellular Ca^2+^ just before the addition of Ca^2+^ mobilizing agonists (1–2 min). Ca^2+^ changes in Jurkat cells loaded with fura 2/AM were measured via rapid alternation of the excitation monochromator between 340 and 380 nm, with fluorescence emission measured at 510 nm using a ratiometric spectrofluorometer (PTI, Lawrenceville, NJ, USA). Cytosolic Ca^2+^ responses are presented as the changes in the fluorescence ratio values measured at 340/380 nm for Fura 2. The data are reported as peak amplitude changes in fluorescence values (ΔF) and as Ca^2+^ release rates using computation of ΔF/second linear initial rates to reflect the maximal Ca^2+^ release rate and presented as the means ± S.E.M., with the number of experimental repetitions indicated in parentheses.

### 4.4. Calcium Small Cell-Number Microscope Photometry Assays

To measure the fluorescence intensity in small cell-number populations, Jurkat cells loaded with fura 2/AM were allowed to adhere to the coverslip for a minimum of 15 min at room temperature to allow sufficient adhesion prior to mounting on the stage of an IX51 Olympus microscope (Olympus, Center Valley, PA USA). Jurkat cells were transfected as described below to achieve SERCA overexpression or knockout; assessment of transfection efficiencies (routinely between 85–95%) and expression verification were accomplished via co-transfection with a GFP-encoded plasmid (pEGFP-C2, Clontech, Mountain View CA, USA). A knife-blade aperture was used to isolate small groups of cells (approximately 30–60 lymphocytes) to record signals from, which could be shown to be expressing GFP using a GFP filter cube (Chroma Technology Bellows Falls, VT USA). Ca^2+^ changes were then determined by switching to a Fura 2 filter cube (Chroma Technology) and directing excitation light (340/380 nm) via a Xenon arc lamp monochromator-based system (PTI, Lawrenceville, NJ, USA) onto Fura-2-loaded cells using a UAPO 40X oil immersion 340 nm transmissible objective (Olympus). Fluorescence emission was directed to a photomultiplier tube-based photon-counting detector for computer collection and processing using the Felix software program (PTI, Version 1.41).

### 4.5. SERCA Overexpression

The overexpression of SERCA2b (SERCA2b_OE_) and SERCA3 (SERCA3_OE_) protein was achieved with Human SERCA2b (pcDNA3.1+) (Plasmid #75188) and Human SERCA3 (pMT2) (Plasmid #75189) from Addgene via electroporation (Neon NxT, Thermo Fisher Scientific) with electroporation parameters (1700 V; 20 ms; 1 pulse). Jurkat T cells, 5 × 10^7^ cells/mL, resuspended in Neon NxT Resuspension R buffer with respective plasmid (100 µg/mL). Electroporation was carried out following manufacturer’s protocols using the 10 μL pipette tip electrode configuration and transfection efficiencies were estimated using co-transfection experiments with a GFP-expressing plasmid (pEGFP-C2, Clontech, Mountain View, CA USA), which matched manufacturer’s protocols and consistently yielded expression levels between 85–95%. Following electroporation, cells were transferred to RPMI 1640 medium; 72 h post-electroporation, cells were utilized for Ca^2+^ assay and protein overexpression by Western blot.

### 4.6. SERCA CRISPR Knockout

The knockout of SERCA2 (SERCA2_KO_) and SERCA3 (SERCA3_KO_) was carried out using Cas9 protein and single guide RNA (sgRNA) (Thermo Fisher Scientific). Cas9 protein (125 μg/mL) and sgRNA (28.2 μg/mL) were combined in Neon NxT resuspension genome editing (GE) buffer and incubated at room temperature to form ribonucleoprotein complexes (Cas9-RNPs). Jurkat T cells, 2 × 10^7^ cells/mL resuspended in GE buffer, were combined with Cas9-RNPs and electroporated with the Neon NxT transfection device (Thermo Fisher Scientific) with electroporation parameters (1700 V; 20 ms; 1 pulse). sgRNA sequences were used as follows: (5′-CUUCGGCGUCAACGAGAGUA-3′) for SERCA2 (ATP2A2) and (5′-AGGAUCAGCAUGAUGACCAG-3′) for SERCA3 (ATP2A3), respectively. Electroporation was carried out as described above for the overexpression experiments and evaluated for transfection efficiency using a GFP-encoded expression vector (pEGFP-C2, Clontech, Mountain View, CA USA). Following electroporation, cells were transferred to and maintained in the RPMI 1640 medium; after 5 days post-electroporation, the knockout efficiency was assessed at protein expression level by Western blot, and cells were utilized for Ca^2+^ assays.

### 4.7. Western Blot Analysis

To evaluate gene overexpression and knockout efficiency, Western blotting was performed using standard methodologies. In brief, after 72 h of Jurkat T cell plasmid electroporation and 5 days of Jurkat T cell CRISPR/Cas9 ribonucleoprotein electroporation, cells were collected from culture flasks and pelleted via centrifugation. After washing with ice-cold phosphate-buffered saline, cell pellets were lysed with ice-cold RIPA buffer (Thermo Fisher) supplemented with 1X Halt protease and phosphatase inhibitor (Thermo Fisher Scientific, Waltham, MA, USA) for 20 min on ice. Whole-cell lysates were clarified by centrifugation (15,000× *g* for 15 min at 4 °C), and total protein concentration was determined by the Pierce BCA assay (Thermo Fisher Scientific, Waltham, MA, USA) according to manufacturer protocols. Samples were analyzed using sodium dodecyl sulfate-polyacrylamide gel (SDS-PAGE) electrophoresis and transferred to low-fluorescent PVDF membranes (Bio-Rad, Hercules, CA, USA) by wet transfer. Membranes were blocked for 1 h at room temperature with intercept blocking buffer (LI-COR, Lincoln, NE, USA) and incubated overnight at 4 °C with primary antibodies: anti-SERCA2 (Santa Cruz Biotechnology, Dallas, TX, USA), anti-SERCA3 (Thermo Fisher Scientific, Waltham, MA, USA), anti-Glut3 and anti-ß Actin (Santa Cruz Biotechnology, Dallas, TX, USA). After treatment with primary antibodies, the membranes were washed four times for 5 min with washing buffer (TBST) and incubated with the IRDye 800CW secondary antibodies (LI-COR, Lincoln, NE, USA) for 1 h at room temperature. The bands of proteins were detected by the LI-COR Odyssey M imaging system using Image Studio software Version 6.0. (Lincoln, NE, USA) ß-Actin was used as loading control for all Western blots. Where necessary, blots were washed once for 5 min with TBST, then stripped for 25 min at room temperature using NewBlot IR Stripping Buffer (LI-COR) with gentle agitation. After stripping, the blots were re-blocked and incubated with primary antibody, followed by incubation with secondary antibody, and finally developed and imaged following the same procedure as before.

### 4.8. Cell Proliferation Assay

The viability of Jurkat cells in proliferation was measured using the CellTiter 96 AQ_ueous_ Non-Radioactive Cell Proliferation Assay (MTS) system (Promega, Madison, WI, USA) according to the manufacturer’s instructions. Briefly, 3 × 10^4^ cells from various SERCA_OE_ or SERCA_KO_ cell populations treated with mitogens or SERCA modulators were plated into each well of a 96-well plate, and at the end of the experiment, 20 μL of MTS reagent was added to the well. After 3.5 h of incubation at 37 °C in a humidified, 5% CO_2_ atmosphere, absorbance at 490 nm was measured using a microplate reader (Spectramax ID3, Molecular Devices, San Jose, CA, USA) to determine the viability of the cells, which was expressed as a percentage of the control group. Three replicate wells per experimental condition were used to obtain measures of cell proliferation.

### 4.9. Glucose Uptake Assay

Glucose uptake in Jurkat cells was measured with the Glucose Uptake-Glo™ Assay according to the manufacturer’s instructions (Promega, Madison, WI, USA). Briefly, cells exposed to desired treatment conditions from various SERCA_OE_ or SERCA_KO_ cell populations were washed with glucose free phosphate-buffered saline (PBS) and incubated with 2-deoxy-d-glucose (2DG) for 10 min. After several additions of buffer and an additional incubation for 1 h, glucose uptake was measured as luminescence using a plate reader (Spectramax ID3, Molecular Devices, San Jose, CA, USA) and reported as counts per second with an integration time of one second.

### 4.10. Glutathione Assay

The ratio of reduced glutathione to oxidized glutathione (GSH/GSSG) in Jurkat cells was measured with the GSH/GSSG-Glo™ Assay according to manufacturer’s instructions (Promega, Madison, WI, USA). Briefly, cells exposed to desired treatment conditions from various SERCA_OE_ or SERCA_KO_ cell populations were washed and diluted with Hank’s balanced salt solution (HBSS) and loaded into wells of an opaque, white 96-well plate. After several additions of buffer and incubation periods, GSH levels were determined as luminescence in the plate reader (Spectramax ID3, Molecular Devices, San Jose, CA, USA) and reported as counts per second with an integration time of one second.

### 4.11. Interleukin-2 (IL-2) Assay

The amount of IL 2 in culture supernatants was measured with the Lumit^®^ IL-2 (Human) Immunoassay according to the manufacturer’s instructions (Promega, Madison, WI, USA). Briefly, cell culture supernatants from SERCA_OE_ or SERCA_KO_ cell populations with or without exposure to PHA were collected after desired treatment and stored at −80 °C until use. After thawing, samples were brought to room temperature and transferred into wells of an opaque, white 96-well plate. Then, 50 μL of a 2x antibody mixture was added to 50 μL of transferred samples or standard dilutions and incubated for 60 min. Following incubation, 25 μL of Lumit™ Detection Reagent was added, and IL 2 levels were determined as luminescence with a plate reader (Spectramax ID3, Molecular Devices, San Jose, CA, USA) and recorded as counts per second with an integration time of one second.

### 4.12. Statistical Analysis

All statistical analyses were performed using GraphPad Prism 10.3.0 (GraphPad Software Inc., San Diego, CA, USA). For Ca^2+^ measurements in the large cell-number and small cell-number experiments, representative responses were compared, and statistical significance was determined using Student’s *t* test. Notably, *p* values ≤ 0.05 were considered to represent significant differences in the results. For lymphocyte proliferation, glucose uptake, GSH and IL-2 experiments, data were analyzed using one-way ANOVA and two-way ANOVA employing Dunnett’s and Tukey’s multiple comparisons test where applicable, with *p* < 0.05 determining significance.

## Data Availability

Dataset available on request from the authors.
